# Prognostic value of neutrophil-to-monocyte/lymphocyte ratio for 28-day mortality in ICU sepsis patients: a retrospective cohort study

**DOI:** 10.3389/fmed.2024.1434922

**Published:** 2024-08-15

**Authors:** Yan Xia, Heping Xu, Jinyuan Xie, Huan Niu, Xiongwei Cai, Feng Zhan, Duoyi Wu, Jinjian Yao

**Affiliations:** ^1^Department of General Practice, The First Affiliated Hospital of Hainan Medical University, Haikou, China; ^2^Department of Emergency Medicine, Hainan General Hospital, Hainan Affiliated Hospital of Hainan Medical University, Haikou, China

**Keywords:** Sepsis, NMLR, 28-day mortality, inflammatory biomarker, MIMIC-IV

## Abstract

**Background:**

Sepsis is a life-threatening condition that requires rapid assessment to reduce mortality. This study investigates the relationship between the Neutrophil-to-Monocyte/Lymphocyte Ratio (NMLR) upon ICU admission and 28-day mortality in sepsis patients.

**Methods:**

A retrospective analysis was performed using clinical data from sepsis patients in the Medical Information Mart for Intensive Care IV (MIMIC-IV). Multivariate logistic regression, sensitivity analyses, and Restricted Cubic Spline (RCS) models were employed to explore the relationship between ICU admission NMLR and 28-day mortality. Kaplan–Meier method and inverse probability weighting (IPW) were used to adjust for confounders and estimate survival outcomes. Receiver operating characteristic (ROC) curve evaluating the predictive value of NLMR for 28-day mortality in ICU sepsis patients. Subgroup analyses considered factors like age, sex, race, comorbidities, and disease severity.

**Results:**

In total, 8,710 patients were included. Increased NMLR was associated with higher 28-day all-cause mortality, confirmed by multiple logistic regression models. In Model 3, after adjusting for confounders, each standard deviation increase in NMLR was associated with a 1.5% increase in 28-day mortality risk. Kaplan–Meier and IPW survival analyses showed higher 28-day all-cause mortality in patients with elevated NMLR levels at ICU admission compared to those with lower levels (*p* < 0.0001, *p* = 0.031). RCS models suggested a potential non-linear relationship between NMLR and 28-day mortality. ROC curve for the NMLR model, with an AUC of 0.658 (95% CI: 0.642–0.673). Sensitivity analyses confirmed the association even after excluding patients with myocardial infarction and severe liver disease.

**Conclusion:**

Elevated NMLR at ICU admission is significantly associated with increased 28-day all-cause mortality in sepsis patients, suggesting its potential as an early prognostic indicator for risk assessment and intervention.

## Introduction

Sepsis is defined as a life-threatening organ dysfunction caused by dysregulated host response to infection (1). In recent years, the incidence and prevalence of sepsis have significantly increased globally (2, 3), constituting a major global health burden. Therefore, rapid detection of sepsis is crucial for reducing mortality. Although much about the pathophysiology of sepsis remains unknown, most studies indicate that it is directly related to the altered immune function and dysregulation of inflammatory and anti-inflammatory systems (4). Many laboratory parameters serve as biomarkers for inflammation and prognosis, but they have limitations that restrict their clinical applications, including high costs, limited diagnostic accuracy, and long processing times. In contrast, inflammation and immune cell counts are inexpensive and easily accessible measurement methods, allowing easy acquisition of inflammation response parameters from full blood cell counts (5).

Many studies have shown that ratios involving different types of white blood cells, such as the Neutrophil-to-Lymphocyte Ratio (NLR) and the Neutrophil-to-Monocyte-to-Lymphocyte Ratio (NMLR), are valuable biomarkers for assessing systemic inflammation and predicting the prognosis of certain health problems (6, 7). The ratio of peripheral neutrophil and monocyte counts to peripheral lymphocyte counts (NMLR) is an effective indicator of inflammation and immune status. Some studies suggest that the NMLR is a prognostic indicator of inflammatory and immune diseases as well as acute myocardial infarction (8–10). However, evidence regarding the correlation between NMLR and 28-day mortality in intensive care unit (ICU) sepsis patients remains limited. Therefore, we conducted this study to investigate the relationship between NMLR at ICU admission and 28-day mortality in ICU sepsis patients.

## Methods

### Database introduction

The data used in our study were extracted from the Medical Information Mart for Intensive Care IV (MIMIC-IV version 2.2) database (DOI: 10.13026/6 mm1-ek67) (11). This registry was developed from sophisticated multi-parameter monitoring systems at the Beth Israel Deaconess Medical Center (BIDMC) in Boston, Massachusetts, encompassing detailed records of over 50,000 patients admitted from 2008 to 2019 (12). Author Xu, having completed the Citi Program online training course (record ID 59568270), extracted data using the PostgreSQL tool.

### Definitions

The NMLR is defined as the ratio of the combined counts of peripheral neutrophils and monocytes to the count of peripheral lymphocytes. It is further categorized into three classes based on tertiles: NMLR1 (<4.28), NMLR2 (4.28–9.39), and NMLR3 (>9.39). Acute kidney injury (AKI) is identified by a serum creatinine increase exceeding 1.5 times the baseline (13). Shock is defined by the need for vasoactive drugs during an ICU stay. Sepsis was identified in accordance with the Sepsis-3 criteria, defined by a Sequential Organ Failure Assessment (SOFA) score of 2 or more points concurrent with an infection (1).

### Participants and data extraction

Patients aged over 18 years and above who were admitted to the Intensive Care Unit and diagnosed with sepsis were enrolled in this retrospective study. Exclusion criteria included: (1): Patients with previous ICU admissions, to avoid data duplications; (2) Patients whose survival time was less than 24 h, ensuring sufficient evaluation of their clinical status and outcomes; (3) Patients with incomplete data regarding neutrophil count, monocyte count, or lymphocyte count, which are critical for precise NMLR calculation.

The primary endpoint of this study was the 28-day mortality rate. The extracted dataset encompassed a range of demographic and clinical variables, including age, gender, ethnicity, medical history of myocardial infarction, congestive heart failure, chronic pulmonary disease, diabetes without controlled, severe liver disease, cerebrovascular disease, renal disease, and the Charlson comorbidity index. Additionally, the study considered the initial SOFA score, Simplified Acute Physiology Score II (SAPS II), vital signs (systolic, diastolic, and mean arterial blood pressures; heart rate; respiratory rate; body temperature; pulse oximetry readings), anthropometric data (weight), and laboratory parameters (white blood cell, hemoglobin, platelet, anion gap, bicarbonate, chloride concentration, glucose, sodium, potassium, creatinine, blood urea nitrogen, calcium, prothrombin time, and albumin). Clinical interventions such as invasive ventilation and continuous renal replacement therapy (CRRT), as well as the occurrence of septic shock and acute kidney injury within the first 2 days after admission, were also recorded. Furthermore, the study tracked the length of stay in the ICU, the total duration of hospitalization, and in-hospital mortality. All baseline data were collected within the initial 24 h of ICU admission.

### Statistical analysis

The data were analyzed using R version 4.2.1 and Stata version 18.0. Continuous variables were reported as mean (standard deviation) or median (interquartile range), and categorical variables were expressed as percentages. The baseline characteristics across various NMLR categories were assessed using the chi-square test for categorical data, one-way analysis of variance for normally distributed continuous data, and the Kruskal-Wallis H test for non-normally distributed data.

This study explored the relationship between NMLR and 28-day all-cause mortality through a multivariate logistic regression analysis. To assess multicollinearity, Variance Inflation Factor (VIF) values were utilized, with those exceeding 10 indicating significant multicollinearity. Three distinct models were developed: Model 1, an unadjusted baseline model; Model 2, which was adjusted for age, sex, and ethnicity; and Model 3, which accounted for all variables using a backward regression approach. The comprehensive set of variables incorporated into the final analysis included age, congestive heart failure, chronic pulmonary disease, diabetes without control, severe liver disease, cerebrovascular disease, renal disease, Charlson comorbidity index, first day of SOFA score, SAPS II, systolic blood pressure, diastolic blood pressure, mean blood pressure, heart rate, respiratory rate, Temperature, pulse oxygen saturation, weight, hemoglobin, anion gap, bicarbonate, chloride, sodium, potassium, creatinine, blood urea nitrogen, calcium, prothrombin time, albumin, Invasive ventilation, Septic shock, AKI within 2 days of admission, length of stay in the ICU, Length of hospital stay. A backward elimination regression procedure was employed to include variables meeting a significance threshold of *p* < 0.05 and to exclude those with significance levels exceeding *p* > 0.1.

Subgroup analyses were conducted based on factors such as age (<65 and ≥ 65 years), sex, ethnicity, myocardial infarction, congestive heart failure, cerebrovascular disease, chronic pulmonary disease, diabetes without control, sever liver disease, renal disease, Charlson comorbidity index (<7 and ≥ 7), AKI, CRRT, invasive ventilation.

Sensitivity analysis was also conducted to further validate our findings. For this purpose, logistic regression analysis was performed on subsets of patients: those excluded due to myocardial infarction and those excluded due to both myocardial infarction and severe liver disease. To mitigate potential confounding factors, weighted inverse probability (IPW) regression analysis was applied to unweighted raw data (14).

A restricted cubic spline was used to determine the cut-off value and visualize the nonlinear relationship between the ICU admission NMLR and 28-day mortality. The 28-day survival rates of patients admitted to ICUs with varying levels of NMLR were compared using Kaplan–Meier analysis and IPW survival analysis (15). Receiver operating characteristic (ROC) curve evaluating the predictive value of NLMR, NLR, and SOFA for 28-day mortality in ICU sepsis patients. A two-sided test was utilized, with a *p*-value of less than 0.05 deemed to indicate statistical significance.

## Results

### Baseline characteristics of the participants

**Figure 1:**
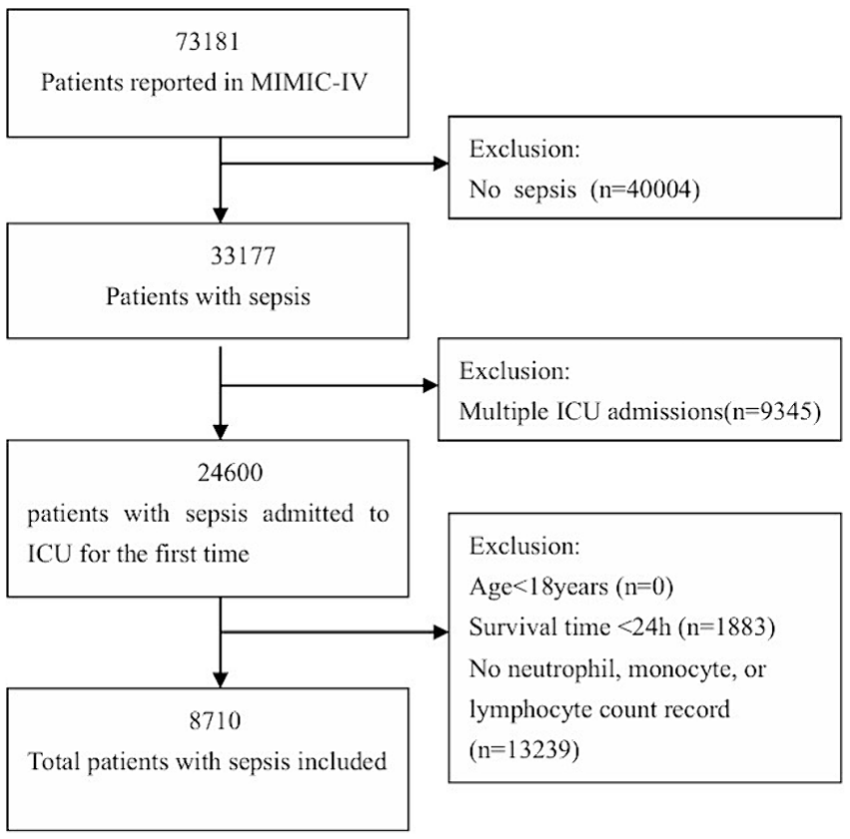
Flow chart of patient selection for analysis.

A total of 8,710 patients from the MIMIC-IV database met the inclusion criteria, excluding 13,239 cases with missing NMLR values, as illustrated in Figure 1. The baseline characteristics of the patients across the NMLR categories are detailed in Table 1. The average age of these patients was 66.3 years with a standard deviation of 16.0, and approximately 57.6% were male. The subjects were further categorized into three classes based on tertiles: NMLR1 (<4.28), NMLR2 (4.28–9.39), and NMLR3 (>9.39). There were no significant differences among the groups in terms of severe liver disease, mean blood pressure, heart rate, and respiratory rate (all *p* > 0.05). The percentage of septic shock, the SOFA score on the first day, and the SAPS II score in the high NMLR group were significantly higher compared to the low NMLR group, indicating that NMLR may be closely related to the severity of sepsis. Notably, the 28-day mortality rate was higher in the group with the highest NMLR compared to the group with a lower NMLR (*p* < 0.001). Additionally, several other factors exhibited statistically significant differences, including myocardial infarction, congestive heart failure, chronic pulmonary disease, uncontrolled diabetes, severe liver disease, cerebrovascular disease, renal disease, Charlson comorbidity index, first-day SOFA score, SAPS II Score, heart rate, respiratory rate, white blood cell count, platelet count, anion gap, potassium levels, creatinine levels, glucose levels, blood urea nitrogen, calcium levels, prothrombin time, albumin levels, invasive ventilation, septic shock, AKI within 2 days of admission, length of ICU stay, and length of hospital stay.

**Table 1:** Baseline characteristic of the sepsis patients.

		NMLR			*P*
Characteristics	All patients	NMLR1 < 4.28	4.28 ≤ NMLR2 < 9.39	NMLR3 ≥ 9.39	
Number	8,710	2,904	2,903	2,903	
Age, years	66.3 (16.0)	63.3 (16.4)	67.1 (15.5)	68.7 (15.7)	0.005
Sex (male, *n*)	5,018 (57.6)	1,592 (54.8)	1733 (59.7)	1,693 (58.3)	0.001
Ethnicity (white, *n*)	5,294 (60.8)	1708 (58.8)	1806 (62.2)	1780 (61.3)	0.023
Myocardial infarction	1,417 (16.3)	402 (13.8)	457 (15.7)	558 (19.2)	<0.0001
Congestive heart failure	2,361 (27.1)	593 (20.4)	807 (27.8)	961 (33.1)	<0.0001
Chronic pulmonary disease	2052 (23.6)	619 (21.3)	671 (23.1)	761 (26.2)	<0.0001
Diabetes without control	1970 (22.6)	628 (21.6)	720 (24.8)	622 (21.4)	0.003
Severe liver disease	547 (6.3)	164 (5.6)	181 (6.2)	202 (7.0)	0.119
Cerebrovascular disease	1,163 (13.4)	357 (12.3)	378 (13.0)	428 (14.7)	0.019
Renal disease	1991 (22.9)	514 (17.7)	675 (23.3)	802 (27.6)	<0.0001
Charlson comorbidity index	5 (4–8)	5 (3–7)	5 (4–8)	6 (4–8)	0.0001
First day of SOFA	6 (4–9)	5 (4–8)	6 (4–9)	7 (4–10)	0.0001
SAPSII	38 (30–48)	35 (27–44)	38 (31–46)	42 (34–51)	0.0001
SBP, mmHg	115.4 (15.1)	115.9 (14.6)	115.5 (15.1)	114.9 (15.5)	0.004
DBP, mmHg	62.4 (10.4)	62.7 (10.0)	62.1 (10.4)	62.3 (10.6)	0.006
MBP, mmHg	77.4 (10.1)	77.8 (9.9)	77.3 (10.2)	77.1 (10.3)	0.056
Heart rate, beats/min	86.7 (16.3)	86.5 (16.2)	85.6 (16.1)	88.2 (16.6)	0.166
Respiratory rate, beats/min	19.8 (3.9)	19.5 (4.0)	19.6 (3.8)	20.3 (3.9)	0.219
Temperature, °C	36.9 (0.53)	36.9 (0.51)	36.9 (0.54)	36.9 (0.54)	0.022
SpO2, %	96.9 (2.2)	97.1 (2.0)	97.0 (2.1)	96.7 (2.5)	<0.0001
Weight, kg	83.3 (24.3)	83.3 (22.6)	84.8 (25.0)	81.9 (25.2)	<0.0001
WBC, cell/mm3	12.1 (8.8–16.3)	11.0 (8.1–14.8)	11.7 (8.7–15.8)	13.7 (10.0–18.5)	0.0001
Hemoglobin, mg/dL	10.3 (8.9–11.9)	10.4 (9.0–11.9)	10.4 (9.0–11.9)	10.2 (8.7–11.9)	0.0117
Platelet, cell/mm3	175 (125–238.5)	166 (118.8–230)	175 (126.5–234)	184.5 (130.5–251.5)	0.0001
Anion gap, mEq/L	15 (12.5–17.5)	14 (12–16.5)	14.5 (12.5–17)	15.5 (13.5–18.5)	0.0001
Bicarbonate, mEq/L	22 (19.5–24.5)	22.5 (20–24.5)	22.5 (20–24.5)	21.5 (19–24)	0.0001
Chloride, mEq/L	104 (100–107)	104.5 (101–108)	104 (100–107)	102.5 (98.5–106.5)	0.0001
Glucose, mg/dL	131.1 (113–161.6)	129 (112.9–155.0)	131.4 (113.5–159.4)	133 (113–171)	0.0001
Sodium, mEq/L	138.5 (136–141)	138.5 (136–141)	138.5 (136–141)	138 (135–141)	0.0001
Potassium, mEq/L	4.2 (3.9–4.65)	4.2 (3.85–4.55)	4.2 (3.9–4.6)	4.25 (3.9–4.75)	0.0001
Creatinine, mg/dL	1.05 (0.8–1.65)	0.95 (0.75–1.35)	1.05 (0.75–1.6)	1.25 (0.85–2.05)	0.0001
BUN, mg/dL	20.5 (14–34.5)	17.5 (12.5–27.5)	20 (14–34)	26 (16.5–43)	0.0001
Calcium, m Eq/L	8.25 (7.85–8.7)	8.25 (7.85–8.7)	8.25 (7.9–8.7)	8.25 (7.8–8.7)	0.0058
PT, sec	14.15 (12.65–16.4)	14.1 (12.55–15.78)	14.15 (12.75–16.5)	14.2 (12.7–17.25)	0.0001
Invasive ventilation	1923 (22.1)	587 (20.2)	612 (21.1)	724 (24.9)	<0.0001
CRRT	892 (10.2)	199 (6.9)	271 (9.3)	422 (14.5)	<0.0001
Vasoactive drug	4,510 (51.8)	1,418 (48.8)	1,529 (52.7)	1,563 (53.8)	<0.0001
Septic shock	4,510 (51.8)	1,418 (48.8)	1,529 (52.7)	1,563 (53.8)	<0.0001
AKI within 2 days of admission	5,938 (68.2)	1864 (64.2)	2008 (69.2)	2066 (71.2)	<0.0001
LOS ICU	3.19 (1.89–6.61)	2.84 (1.71–5.75)	3.21 (1.88–6.54)	3.75 (2.08–7.51)	0.0001
LOS hospital	8.97 (5.63–15.80)	8.31 (5.30–14.80)	8.98 (5.74–15.7)	9.78 (5.85–16.83)	0.0001
In-hospital mortality	1,251 (14.4)	224 (7.7)	360 (12.4)	667 (23.0)	<0.0001
28-day mortality	1,506 (17.3)	267 (9.2)	446 (15.4)	793 (27.3)	<0.0001

Continuous variables are presented as means (SDs) or medians (quartiles), while categorical variables are presented as absolute numbers (percentages). SBP, systolic blood pressure; DBP, diastolic blood pressure; MBP, mean blood pressure; SpO_2_, pulse oxygen saturation; SOFA, Sequential Organ Failure Assessment score; SAPS II, Simplified Acute Physiology Score II; WBC, white blood cell; BUN, blood urea nitrogen; RRT, renal replacement therapy; AKI, acute kidney injury; NMLR, (neutrophil + monocyte)/lymphocyte ratio; LOS, length of stay.

### Association of NMLR with 28-day mortality

The relationship between NMLR and 28-day mortality is shown in Table 2. NMLR was divided into three groups. Three distinct logistic regression models were developed to evaluate the independent effect of NMLR on 28-day all-cause mortality among ICU patients with sepsis. Logistic regression analysis indicated a positive relationship between NMLR and the risk of 28-day all-cause mortality, with an unadjusted odds ratio (OR) of 1.029 (95% CI 1.026–1.033). Using NMLR1 as the reference, the crude ORs for NMLR2 and NMLR3 groups were 1.793 (95% CI 1.526–2.107) and 3.712 (95% CI 3.195–4.313), respectively. After adjusting for age, sex, and ethnicity, the positive association persisted (OR 1.028; 95% CI 1.024–1.032), with adjusted ORs for NMLR2 and NMLR3 at 1.685 (95% CI 1.431–1.984) and 3.396 (95% CI 2.916–3.955), respectively. Model 3, which incorporated adjustments for age, congestive heart failure, chronic pulmonary disease, uncontrolled diabetes, severe liver disease, cerebrovascular disease, renal disease, Charlson comorbidity index, first-day SOFA score, SAPS II Score, systolic blood pressure, diastolic blood pressure, mean blood pressure, heart rate, respiratory rate, temperature, pulse oxygen saturation, weight, hemoglobin, anion gap, bicarbonate, chloride, sodium, potassium, creatinine, blood urea nitrogen, calcium, prothrombin time, albumin, invasive ventilation, septic shock, AKI within 2 days of admission, length of ICU stay, and length of hospital stay, showed that NMLR maintained a strong positive correlation with 28-day all-cause mortality (OR 1.015; 95% CI 1.010–1.020). The ORs for NMLR2 and NMLR3 in this model were 1.448 (95% CI 1.177–1.784) and 1.962(95% CI 1.607–2.401), respectively. In a multifactorial logistic regression analysis model, the risk of 28-day mortality increased by 1.5% for each 1 SD increase in NMLR.

**Table 2:** Relationship between NMLR and 28-day all-cause mortality in different models.

	Model 1		Model 2		Model 3	
	OR (95% CI)	*p* value	OR (95% CI)	*p* value	OR (95% CI)	*p* value
NMLR	1.029 (1.026,1.033)	<0.0001	1.028 (1.024,1.032)	<0.0001	1.015 (1.010,1.020)	<0.0001
Tertile						
NMLR1	Ref		Ref		Ref	
NMLR2	1.793 (1.526,2.107)	<0.0001	1.685 (1.431,1.984)	<0.0001	1.448 (1.177,1.784)	<0.0001
NMLR3	3.712 (3.195,4.313)	<0.0001	3.396 (2.916,3.955)	<0.0001	1.962 (1.607,2.401)	<0.0001
*p* for trend	<0.0001		<0.0001		<0.0001	

OR, odds ratio; CI, confidence interval; Ref, reference; Model 1 was not adjusted; Model 2 was adjusted for age, sex; and ethnicity Model 3 was adjusted for age, congestive heart failure, chronic pulmonary disease, diabetes without control, severe liver disease, cerebrovascular disease, renal disease, Charlson comorbidity index, first day of Sequential Organ Failure Assessment score, Simplified Acute Physiology Score II, systolic blood pressure, diastolic blood pressure, mean blood pressure, heart rate, respiratory rate, Temperature, pulse oxygen saturation, weight, hemoglobin, anion gap, bicarbonate, chloride, sodium, potassium, creatinine, blood urea nitrogen, calcium, prothrombin time, albumin, Invasive ventilation, Septic shock, acute kidney injury within 2 days of admission, length of stay in the ICU, Length of hospital stay.

**Figure 2:**
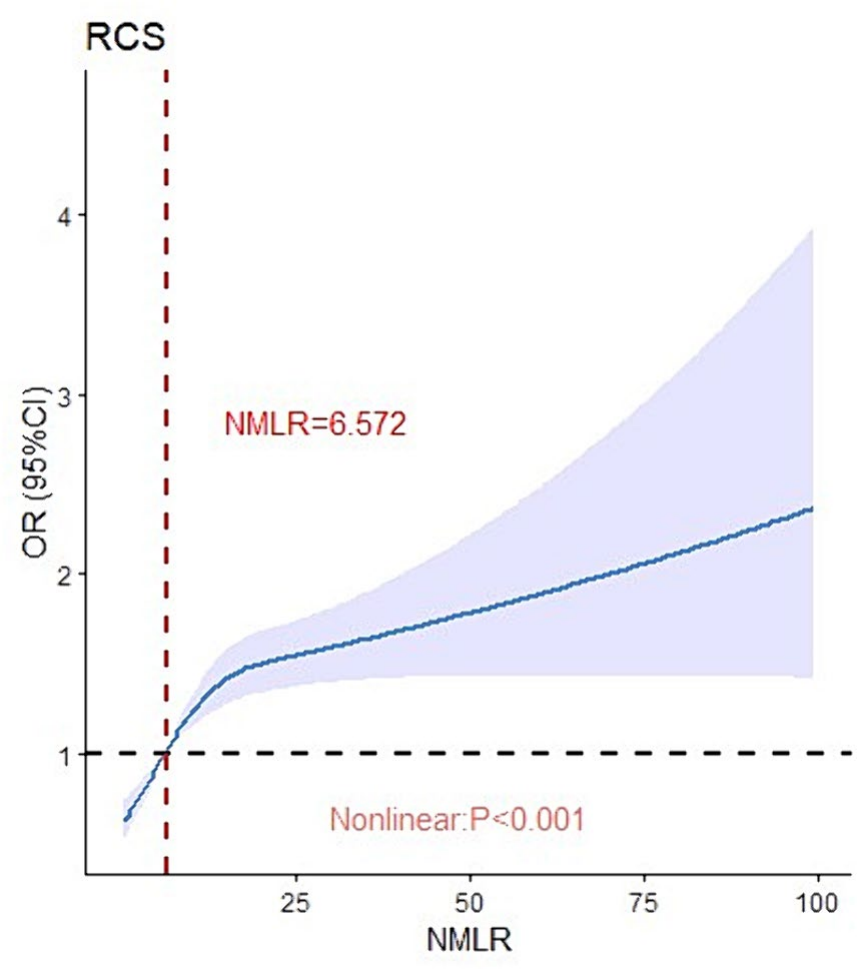
Nonlinear relationship between the NMLR and 28-day mortality.

Critical values were determined using restricted cubic splines to visualize the non-linear relationship between ICU admission NMLR and 28-day mortality. As shown in Figure 2, ICU admission NMLR was nonlinearly associated with 28-day mortality in sepsis patients (*p* < 0.0001). When the NMLR at admission exceeded 6.572, the odds ratio increased rapidly, although the rate of increase diminished as NMLR continued to rise. Overall, the 28-day mortality OR increased with higher levels of ICU admission NMLR.

**Figure 3:**
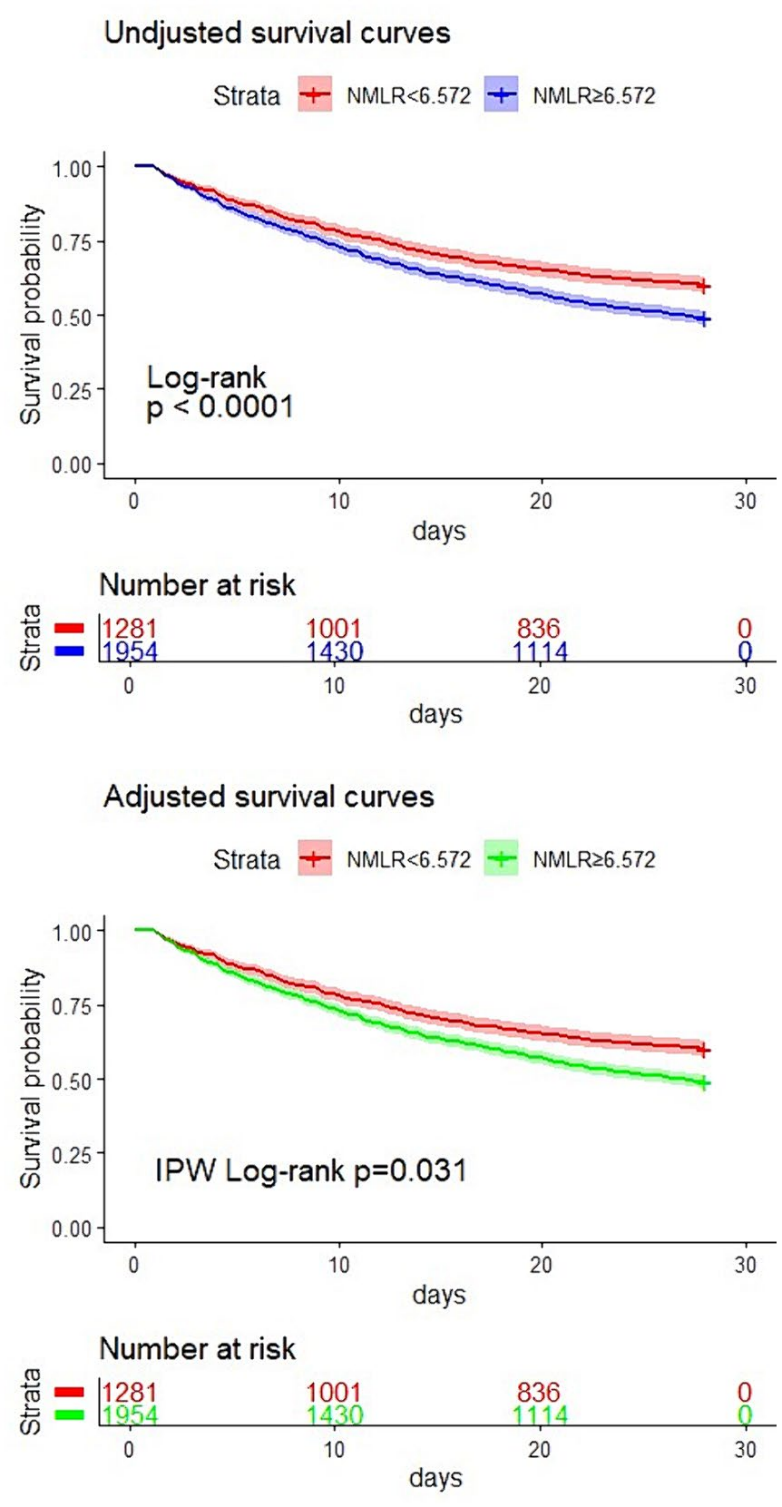
Kaplan–Meier plots for 28-day mortality by ICU admission NMLR strata.

Subsequently, we divided the study population into two groups based on the NMLR cutoff point: a higher NMLR group (NMLR≥6.572) and a lower NMLR group (NMLR<6.572). Kaplan–Meier analysis was then conducted for both groups. As depicted in Figure 3, the survival curve for the higher NMLR group admitted to the ICU was significantly lower than that of the lower NMLR group (log-rank test, *p* < 0.0001). This result was further corroborated by a survival analysis adjusted for confounders using inverse probability weighting (*p* = 0.031). Therefore, a higher NMLR at ICU admission was associated with increased 28-day mortality.

**Figure 4:**
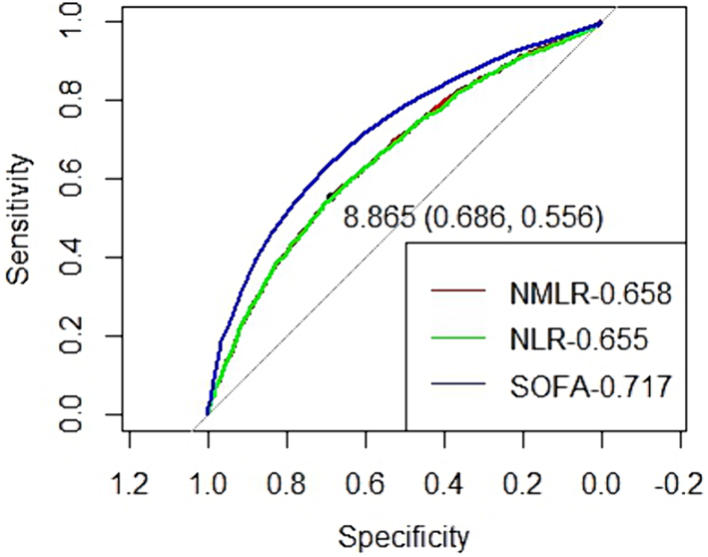
Receiver operating characteristic curve to evaluate the predictive value of NLMR, NLR and SOFA for 28-day mortality of septic patients in ICU.

We performed a receiver operating characteristic (ROC) curve analysis to evaluate the predictive value of NMLR for 28-day mortality in sepsis patients admitted to the ICU and compared it with the performance of NLR and SOFA (Figure 4). The AUC of the NMLR model was 0.658 (95% CI: 0.642–0.673), which was significantly higher than that of the NLR model (0.655, 95% CI: 0.640–0.671; *p* = 0.006), but lower than the AUC of the SOFA model (0.717, 95% CI: 0.703–0.732; *p* < 0.0001). Thus, NMLR at admission has a good predictive value for 28-day mortality in sepsis patients.

### Subgroup analyses

**Figure 5:**
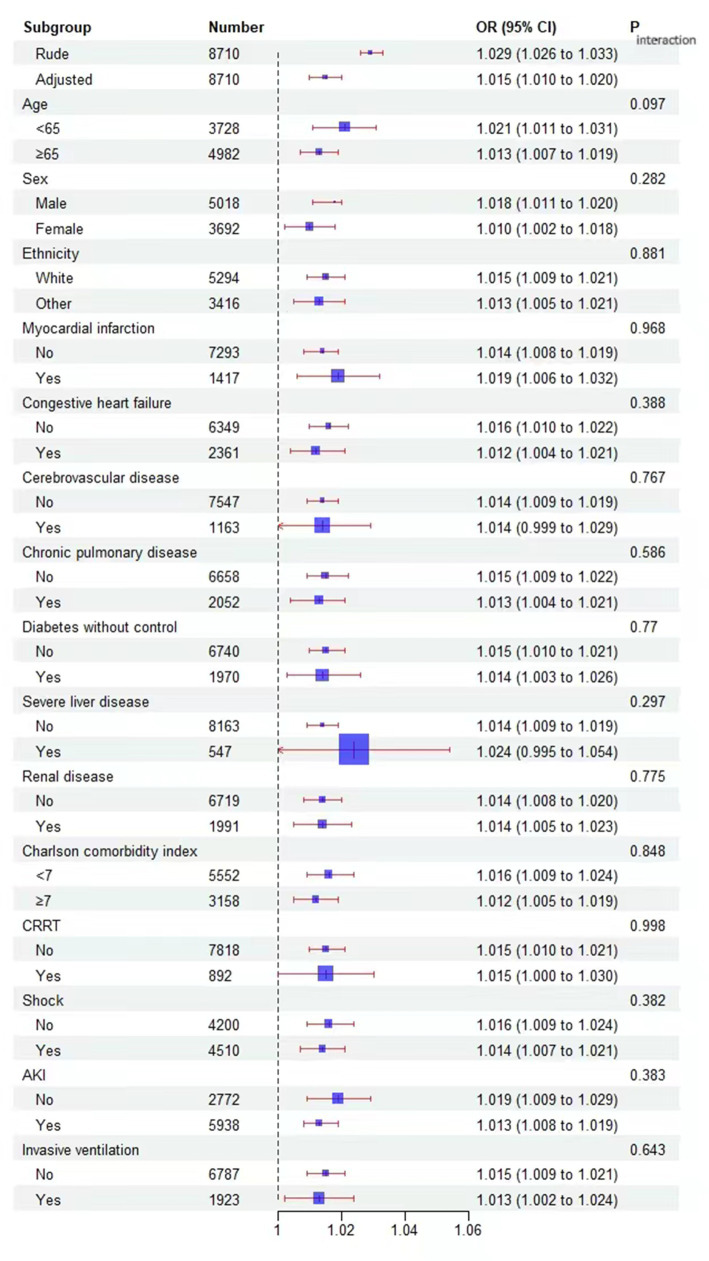
Effect size of NMLR on 28-day mortality in prespecified and exploratory subgroups. The effect size was adjusted for age, congestive heart failure, chronic pulmonary disease, diabetes without control, severe liver disease, cerebrovascular disease, renal disease, Charlson comorbidity index, first day of Sequential Organ Failure Assessment score, Simplified Acute Physiology Score II, systolic blood pressure, diastolic blood pressure, mean blood pressure, heart rate, respiratory rate, Temperature, pulse oxygen saturation, weight, hemoglobin, anion gap, bicarbonate, chloride, sodium, potassium, creatinine, blood urea nitrogen, calcium, prothrombin time, albumin, Invasive ventilation, Septic shock, acute kidney injury within 2 days of admission, length of stay in the ICU, Length of hospital stay., except for the subgroup variable.

To investigate potential clinical heterogeneity, we conducted interaction and stratification analyses (Figure 5). We assessed the relationship between NMLR and 28-day mortality across various subgroups. No significant interactions or differences were observed in the stratified analyses based on age (<65 and ≥ 65 years), sex, ethnicity, myocardial infarction, congestive heart failure, cerebrovascular disease, chronic pulmonary disease, uncontrolled diabetes, severe liver disease, renal disease, Charlson comorbidity index (<7 and ≥ 7), AKI, CRRT, and invasive ventilation.

### Sensitivity analysis

The sensitivity analysis results are shown in Table 3. Using NMLR1 as the reference group, after excluding patients with myocardial infarction, the ORs for the NMLR2 and NMLR3 groups were 1.433 (95% CI: 1.142–1.801) and 2.009 (95% CI: 1.613–2.508), respectively. Further excluding individuals with severe liver disease in addition to myocardial infarction, the ORs for the NMLR2 and NMLR3 groups were 1.415 (95% CI: 1.112–1.804) and 1.952 (95% CI: 1.548–2.468), respectively. After applying inverse probability weighting (IPW) adjustments, the ORs for the NMLR2 and NMLR3 groups were 1.378 (95% CI: 1.238–1.535) and 2.191 (95% CI: 1.939–2.478). The trend test also showed statistical significance (*p* < 0.001).

**Table 3:** Sensitivity analyses.

NMLR	OR (95%CI)	*p*	*p* for trend
Excluding participants with myocardial infarction			
NMLR1	Ref		
NMLR2	1.433 (1.142 ~ 1.801)	0.0019	
NMLR3	2.009 (1.613 ~ 2.508)	<0.001	<0.001
Excluding participants with myocardial infarction and sever liver disease			
NMLR1	Ref		
NMLR2	1.415 (1.112 ~ 1.804)	0.0049	
NMLR3	1.952 (1.548 ~ 2.468)	<0.001	<0.001
Inverse probability of weighting			
NMLR1	Ref		
NMLR2	1.378 (1.238 ~ 1.535)	<0.001	
NMLR3	2.191 (1.939 ~ 2.478)	<0.001	<0.001

Adjusted for age, congestive heart failure, chronic pulmonary disease, diabetes without control, severe liver disease, cerebrovascular disease, renal disease, Charlson comorbidity index, first day of Sequential Organ Failure Assessment score, Simplified Acute Physiology Score II, systolic blood pressure, diastolic blood pressure, mean blood pressure, heart rate, respiratory rate, Temperature, pulse oxygen saturation, weight, hemoglobin, anion gap, bicarbonate, chloride, sodium, potassium, creatinine, blood urea nitrogen, calcium, prothrombin time, albumin, Invasive ventilation, Septic shock, acute kidney injury within 2 days of admission, length of stay in the ICU, Length of hospital stay.

## Discussion

In this retrospective cohort study, we found that higher NMLR values were independently associated with an increased risk of 28-day all-cause death in ICU sepsis patients. Additionally, subgroup analysis, sensitivity analysis, and survival analysis supported consistent findings.

Sepsis is a systemic inflammatory response syndrome caused by infection. Its pathogenesis is complex and involves multiple biological processes and systems. The primary mechanisms include dysregulation of the inflammatory response, wherein monocytes, neutrophils, and other innate immune cells release pro-inflammatory cytokines, leading to systemic injury (16). In the later stages of sepsis, an excessively active immune response may shift to an immunosuppressive state, characterized by lymphocytopenia, proinflammatory responses of monocytes and macrophages (17), and neutrophil dysfunction. This increases the release and activation of immunosuppressive bone marrow-derived suppressor cells (18), which coordinate mediated immunosuppression by releasing anti-inflammatory factors such as interleukin-10 (19). Therefore, comprehensive indices reflecting the activation state of immune cells have significant predictive value for the prognosis of sepsis patients.

As an indicator of inflammation and immune status, the NMLR has been extensively studied for its prognostic value in various diseases, including hepatocellular carcinoma (20), acute myocardial infarction (9, 10), and multiple myeloma (8). However, there have been limited studies examining the relevance of NMLR in ICU sepsis patients. To date, only one study from 2023 has verified the relationship between NMLR and 30-day mortality from sepsis, showing that NMLR levels were significantly higher in deceased patients compared to survivors (21).

This study utilized multivariable logistic regression analysis and restricted cubic splines (RCS) to establish a positive, nonlinear relationship between the NMLR and 28-day all-cause mortality. This association remained significant even after adjusting for various confounding variables such as age, sex, race, and comorbidities. This indicates that NMLR, to some extent, is independent of other clinical factors and can serve as a reliable and independent prognostic marker. Furthermore, both Kaplan–Meier survival analysis and Inverse Probability Weighted (IPW) survival analysis demonstrated that patients with high NMLR had significantly lower 28-day survival rates compared to those with low NMLR. We validated the effectiveness of NMLR as a prognostic indicator from multiple perspectives. Previous studies (22, 23) have identified the heterogeneity of sepsis, which is partly responsible for the lack of precise treatments. However, our subgroup analysis revealed that the association between NMLR and 28-day mortality was consistent across different age groups (<65 years and ≥ 65 years), sexes, and races. This consistency may be due to the underlying pathophysiological mechanisms that are common across these demographics. Comorbid conditions associated with sepsis, including myocardial infarction, congestive heart failure, diabetes, cerebrovascular disease, chronic pulmonary disease, severe liver disease, and renal disease, are major causes of mortality in septic patients. Both sepsis and acute kidney injury (AKI) frequently occur among ICU patients and are closely linked to poor outcomes (24, 25). Within these subgroups, the relationship between NMLR and mortality remained stable. Sensitivity analyses also showed that the association between NMLR and 28-day mortality remained significant even after excluding patients with myocardial infarction and severe liver disease or when applying inverse probability weighting to adjust for confounding factors. These findings further reinforce the robustness of the study’s conclusions.

NLR is a commonly used marker for inflammation and immune response, showing a positive correlation with important sepsis-related scoring systems such as SOFA. It has been proven in multiple studies to be associated with sepsis outcomes (26, 27). To assess the predictive value of NMLR for 28-day mortality in ICU sepsis patients, we conducted a receiver operating characteristic (ROC) curve analysis and compared it with the predictive performance of NLR and SOFA. Our analysis revealed that the AUC of the NMLR model was 0.658 (95% CI: 0.642–0.673), significantly higher than that of the NLR model but lower than the AUC of the SOFA model, which was 0.717 (95% CI: 0.703–0.732). This indicates that NMLR has good predictive value for 28-day mortality in ICU sepsis patients. These findings suggest that NMLR can serve as a reliable prognostic indicator, and future research should explore the feasibility of combining NMLR with other sepsis-related assessment indicators to enhance predictive accuracy.

In conclusion, this study found that a higher NMLR is independently associated with an increased risk of 28-day all-cause mortality in ICU sepsis patients. NMLR can assist clinicians in early identification of high-risk patients, improving prognostic assessment, and guiding anti-inflammatory and immunomodulatory treatment strategies. The mechanisms underlying this association may involve dysregulated inflammatory responses, immune suppression, dynamic changes in immune cells, and systemic inflammatory response syndrome. As a composite indicator, NMLR reflects the inflammatory and immune status across multiple systems, providing a more comprehensive prognostic evaluation. These findings offer new insights for clinical practice.

While this study provides important insights into NMLR as a prognostic indicator for ICU sepsis patients, there are some limitations. First, the study used retrospective data from a single database, which may introduce selection bias and confounding bias. Second, the laboratory data used were collected on the first day of ICU admission, preventing analysis of continuous changes in NMLR. Moreover, as the data in the MIMIC-IV database comes from a single center, it may not fully represent other medical institutions. As Zhang et al. (28) noted, correlation does not directly imply causation; therefore, future studies need to be conducted on a multi-center and large sample basis to enhance the generalizability and external validity of the findings.

## Conclusion

This cohort study showed that an increase in NMLR was associated with an increased risk of 28-day all-cause mortality in patients with sepsis in the ICU. NMLR can serve as a potential biomarker for the prognosis of sepsis patients, aiding clinicians in early risk assessment and intervention.

## Data Availability

The raw data supporting the conclusions of this article will be made available by the authors, without undue reservation.
